# Three QTL from *Oryza meridionalis* Could Improve Panicle Architecture in Asian Cultivated Rice

**DOI:** 10.1186/s12284-023-00640-5

**Published:** 2023-05-02

**Authors:** Ying Yang, Yu Zhang, Jing Li, Peng Xu, Zhijuan Wu, Xianneng Deng, Qiuhong Pu, Yonggang Lv, Walid Hassan Ali Soliman Elgamal, Sheikh Maniruzzaman, Wei Deng, Jiawu Zhou, Dayun Tao

**Affiliations:** 1 Yunnan Key Laboratory for Rice Genetic Improvement, Food Crops Research Institute, Yunnan Academy of Agricultural Sciences, Kunming, 650200 China; 2 Key Laboratory of Tropical Plant Resources and Sustainable Use, Xishuangbanna Tropical Botanical Garden, Chinese Academy of Sciences, Kunming, 650233 China; 3 School of Life Sciences, Yunnan University, Kunming, 650091 China; 4 Rice Research Department, Field Crops Research Institute, Agricultural Research Center, Sakha, 33717 Egypt; 5 Plant Breeding Division, Bangladesh Rice Research Institute, Gazipur, 1701 Bangladesh

**Keywords:** Rice, Panicle architecture, *Oryza meridionalis*, QTL mapping, Near isogenic line (NIL)

## Abstract

Rice panicle architecture is directly associated with grain yield and is also the key target in high-yield rice breeding program. In this study, three BC_6_F_2_ segregation populations derived from the crosses between two accessions of *Oryza meridionalis* and a *O. sativa* spp. *japonica* cultivar Dianjingyou 1, were employed to map QTL for panicle architecture. Three QTL, *EP4.2*, *DEP7* and *DEP8* were identified and validated using substitution mapping strategy on chromosome 4, 9 and 8, respectively. The three QTL showed pleiotropic phenotype on panicle length (PL), grain number per panicle (GNPP), number of primary branches (NPB), number of secondary branches (NSB), and grain width. *DEP7* and *DEP8* showed yield-enhancing potential by increasing GNPP, NPB and NSB, while *EP4.2* exhibited wide grain, short stalk and panicle which can improve plant and panicle architecture, too. Moreover, epistatic interaction for PL was detected between *EP4.2* and *DEP7*, and epistatic analysis between *DEP7* and *DEP8* for GNPP and NPB also revealed significant two QTL interactions. The result would help us understand the molecular basis of panicle architecture and lay the foundation for using these three QTL in rice breeding.

**Supplementary Information:**

The online version contains supplementary material available at 10.1186/s12284-023-00640-5.

## Background

Rice (*Oryza sativa* L.) is one of the most important staple crops in the world; it provides food for more than half of the global population. Under the situation that world population continues to increase and the available arable land area decreases, improving the rice yield is vital to ensure food security (Wang et al. 2018). Rice panicle architecture is directly associated with grain yield and is the key target in high-yield rice breeding, which comprehensively coordinates GNPP, NPB, NSB, PL and grain shape (Itoh et al. 2005). These traits are inherited in a quantitative manner and generally controlled by a number of major and minor quantitative trait loci (QTL).

Over the past two decades, several genes or QTL involved in panicle architecture development were identified from natural variation populations, including *Gn1a* (Ashikari et al. 2005), *IPA1* (Jiao et al. 2010), *DEP1* (Huang et al. 2009), *GNP1* (Wu et al. 2016), *TAW1* (Yuan et al. 2021), *GS5* (Li et al. 2011). For example, *Gn1a* encodes a cytokinin oxidase/dehydrogenase (OsCKX2) that regulates the phytohormone cytokinin. Decreased expression of *OsCKX2* leads to increase the GNPP through cytokinin accumulation in inflorescence meristem (Ashikari et al. 2005). *IPA1* is a semidominant quantitative trait locus, which regulates plant architecture and increases rice grain yield. The *IPA1* encodes OsSPL14 (Souamosa Promoter Binding Protein-Like 14) regulated by microRNA OsmiR156 in vivo. A point mutation of *OsSPL14* results in a reduction in tiller number, grain number (Jiao et al. 2010). *DEP1*, a dominant gene responsible for erect panicle, encodes a phosphatidylethanolamine-binding-protein-like domain protein, which enhanced meristematic activity and reduced inflorescence internode length leading to increased GNPP, NPB and NSB, that results in the enhancement of rice yield (Huang et al. 2009). Such studies have greatly improved our understanding of the genetic control of panicle architecture traits and provided gene resources for breeding application. However, only a few favorable genes can be used for breeding improvement, and the options of using favorable genes in breeding practice are extremely limited. It is very important to explore new gene resources for rice breeding.

The genetic diversity was gradually reduced in the process of domestication from *O. rufipogon* to Asian cultivated rice (Huang et al. 2012; Chen et al. 2019). Narrow genetic basis of Asian cultivated rice results in the yield bottleneck in rice breeding (Tanksley and Mccouch 1997). Interspecific hybridization-introgression played important driving roles in rice domestication and diversification (Zhou et al. 2022). Therefore, enormous effort has been devoted for exploration favorable genes from wild relatives for rice genetic improvement; some QTL responsible for panicle architecture were detected in the past decades (Gaikwad et al. 2020). *O. meridionalis*, originating from the north Australia, the wild relative species as the most distant of the AA genome species from *O. sativa*, conserves a lot of useful traits such as elongation ability, drought avoidance, iron tolerance, salinity tolerance, blast resistance, high amylose starch content (Khush 1997; Zhu and Ge 2005; Mondal 2018; Yichie et al. 2018; Fujino et al. 2019; Wairich et al. 2021). Thus, it was considered that *O. meridionalis* was a valuable genetic resource for Asian cultivated rice improvement.

In this study, two accessions of *O. meridionalis* were used as donors to develop introgression lines (ILs) in a *japonica* variety Dianjingyou 1 (DJY1) background, three ILs with dense panicle architecture were developed by phenotype screening. Three QTL for panicle architecture were identified and validated on chromosome 4, 9, and 8, respectively. Genetic effect analysis indicated that these three QTL showed pleiotropism effect on plant height (PH), panicle architecture and yield related traits. Interaction analysis among the three QTL suggested that *EP4.2* and *DEP7* showed epistatic interaction effect on PL, *DEP7* and *DEP8* showed epistatic interaction effect on GNPP and NPB. These results not only shed more lights on the genetic basis of panicle architecture, but also provided important gene resources for molecular breeding in rice improvement.

## Results

### Characterization of the Recurrent Parent and ILs

**Fig. 1:**
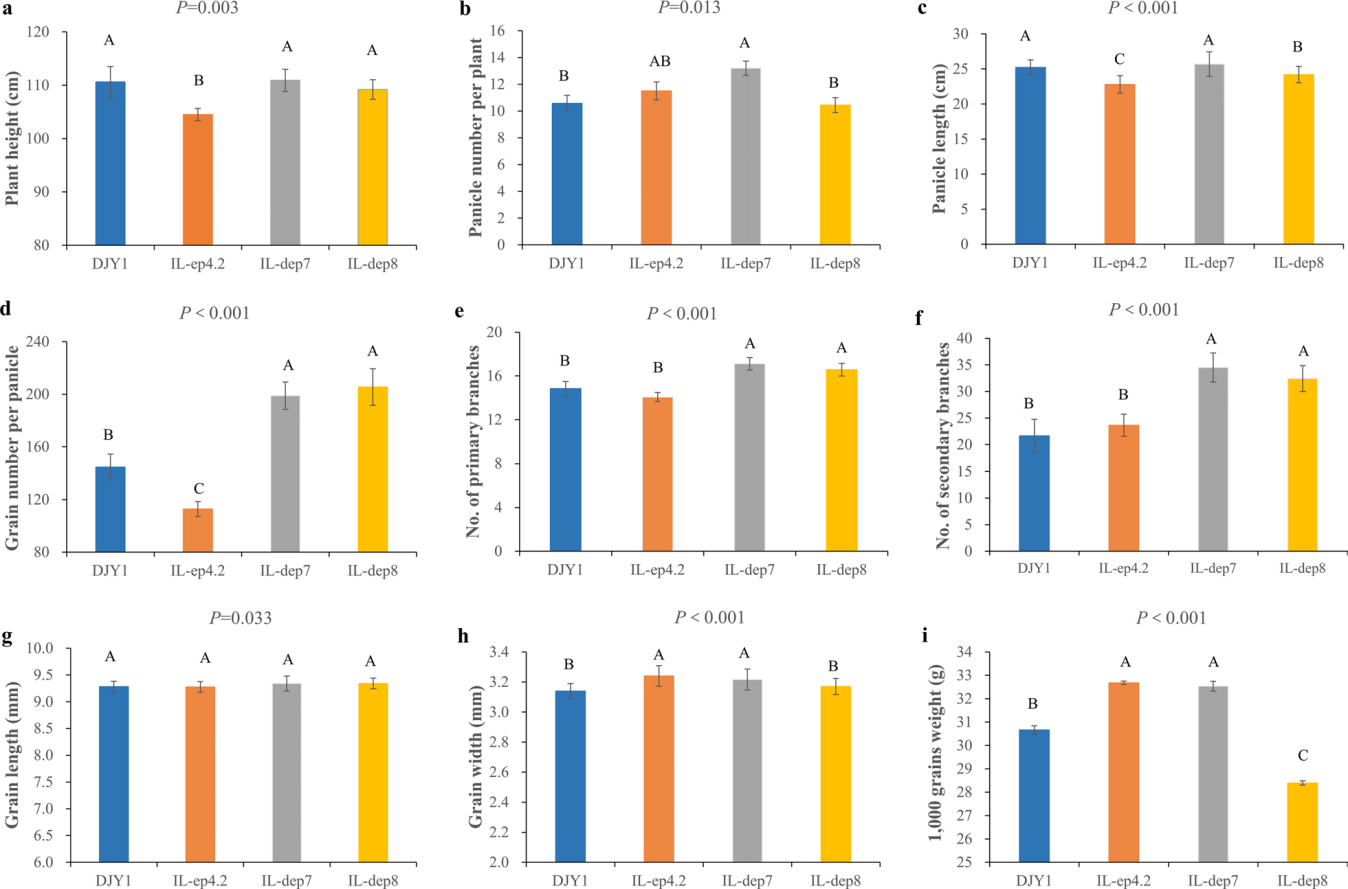
Comparison of phenotype between DJY1 and ILs. **a** Plant height (cm).** b** Panicle number per plant.** c** Panicle length (cm).** d** Grain number per panicle.** e** Number of primary branches.** f** Number of secondary branches.** g** Grain length (mm).** h** Grain width (mm).** i** 1,000 grains weight (g). All multiple comparisons were made at the 1% level

Compared with the recurrent parent DJY1, the IL-*ep4.2* showed shorter stature plant architecture (Fig. 1a), and displayed significant decrease in panicle length (PL) and grain number per panicle (GNPP), whereas grain width (GW) of IL-*ep4.2* was significantly wider than those of DJY1 (Fig. 1c, d). The recurrent parent DJY1 did not differ from IL-*dep7* with respect to plant height (PH), PL and grain length (GL), but panicle number per plant (PN), GNPP, NPB, NSB GW and 1,000 grains weight (TGW) of IL-*dep7* were significantly higher than those of DJY1 (Fig. 1b, d, e, f, h, i). Compared with the recurrent parent DJY1, the IL-*dep8* displayed significant increase in the NPB, NSB, together with GNPP (Fig. 1d, e, f). Furthermore, PL of IL-*dep8* was significantly shorter than that of DJY1 (Fig. 1c). In addition, there was no significant difference in PH between DJY1 and IL-*dep8* (Fig. 1a).

### Detection of QTL for Panicle Architecture Traits

Four hundred and four SSR markers spreading over the whole genome of rice were used to detect the polymorphism between DJY1 and ILs. Out of these, 6 polymorphic markers on chromosome 4, 10 polymorphic markers on chromosome 9 and 12 polymorphic markers on chromosome 8 were detected between DJY1 and IL-*ep4.2*, IL-*dep7*, IL-*dep8*, respectively. No polymorphic markers were detected on the other chromosomes, indicating that only 15.5 cM segment on chromosome 4, 15.5 cM segment on chromosome 9 and 17.9 cM segment on chromosome 8 were substituted by *O. meridionalis* genome in the three ILs, respectively. These polymorphic markers were used for genotyping the three BC_6_F_2_ segregation populations developed by the hybridization between DJY1 and its ILs.

Seven agronomic traits, PL, GNPP, spikelet density (SD), NPB, NSB, GL and GW of the three BC_6_F_2_ segregation populations from crosses between DJY1 and ILs were investigated. Most traits of the BC_6_F_2_ populations exhibited an continuous and normal distributions (Additional file 1: Figure S1, Additional file 2: Figure S2, Additional file 3: Figure S3), whereas the NPB and NSB in the population from the cross between IL-*dep7* and DJY1 (2016H2E273) showed a continuous bimodal distribution (Additional file 2: Figure S2).

To explore the correlations between these traits, we analyzed the Pearson correlation coefficient of three BC_6_F_2_ populations using R 3.5.3 (R package corrplot). SD, NPB and NSB displayed significantly positive correlation with GNPP, which interestingly, were all observed in all three populations, GW also had highly positive correlation with GNPP in 2016H2E273 population (Additional file 4: Figure S4).

To explore favorable alleles for panicle architecture traits, QTL mapping was performed based on phenotype and genotype data. In the population from the cross between IL-*ep4.2* and DJY1 (2016H3E3180), a QTL for PL, GNPP, NPB and GW were identified in the interval between RM1018 and RM17377 on chromosome 4. Compared with the DJY1 allele, the *O. meridionalis* allele increased GW by 0.18 mm and reduced PL, GNPP and NPB by 1.43 cm, 7.46 grains and 0.83 branches, respectively. The QTL explained 40.42%, 6.99%, 28.56% and 66.32% of phenotypic variation for PL, GNPP, NPB and GW, respectively. In the population from the cross between IL-*dep8* and DJY1 (2016H3E3182), a QTL for GNPP, SD, NPB and NSB were detected in the region between RM152 and RM5428 on chromosome 8. Compared with the DJY1 allele, the *O. meridionalis* allele increased GNPP, SD, NPB and NSB by 12.81 grains, 0.63 spikelets per 1 cm panicle, 0.96 and 2.29 branches, respectively. This QTL accounted for 8.05%, 14.18%, 18.11% and 6.13% of the phenotypic variation. In the population from the cross between IL-*dep7* and DJY1 (2016H2E273), the QTL for GNPP, SD, NPB and NSB were detected in the interval between RM201 and RM24718 on chromosome 9. The *O. meridionalis* allele conferred increased GNPP, SD, NPB and NSB by 12.07 grains, 0.24 spikelets per 1 cm panicle, 2.02 and 4.44 branches, respectively. This QTL explain 3.39%, 5.71%, 57.31% and 26.05% of phenotypic variation for GNPP, SD, NPB and NSB, respectively (Table 1). Those three QTL for panicle architecture traits were designated as *EP4.2*, *DEP7* and *DEP8,* respectively.

**Table 1:** Identification of QTL from *O. meridionalis* in the three BC_6_F_2_ populations

Population	Accession	Traits	Chr	Interval	LOD	A	D	PVE	QTL
2016H3E3180	Acc.105279	PL	4	RM1018-RM17377	32.49	−1.43	0.44	40.42	*EP4.2*
		GNPP	4	RM1018-RM17377	4.64	−7.46	4.93	6.99	
		NPB	4	RM1018-RM17377	21.00	−0.83	0.32	28.56	
		GW	4	RM1018-RM17377	69.64	0.16	0.02	66.32	
2016H3E3182	Acc.105279	GNPP	8	RM152-RM5428	3.30	12.81	−4.73	8.05	*DEP8*
		SD	8	RM152-RM5428	8.88	0.63	−0.19	14.18	
		NPB	8	RM152-RM5428	11.08	0.96	−0.01	18.11	
		NSB	8	RM152-RM5428	2.46	2.29	1.13	6.13	
2016H2E273	Acc.104498	GNPP	9	RM201-RM24718	2.38	12.07	7.8	3.39	*DEP7*
		SD	9	RM201-RM24718	2.41	0.49	0.24	5.71	
		NPB	9	RM201-RM24718	37.50	3.16	2.02	57.31	
		NSB	9	RM201-RM24718	13.59	6.95	4.44	26.05	

PL, panicle length; GNPP, grain number per panicle; SD, spikelet density; NPB, the number of primary branch; NSB, the number of secondary branch; GW, grain width. A, additive effect from the *O. merilionalis* allele; D, dominant effect on the *O. merilionalis* allele; PVE, the percentage of phenotypic variance explained by the QTL

### Validation and Delimitation of QTL by Substitution Mapping Method

The method of substitution mapping was used for QTL validation. Individuals from BC_6_F_2_ populations with different recombination length segemnts of the QTL region were selected by marker assisted selection. Then, 6, 4 and 6 homozygous substitution lines were developed by continuous selfing for validation and delimitation for *EP4.2*, *DEP7* and *DEP8*, respectively.

**Fig. 2:**
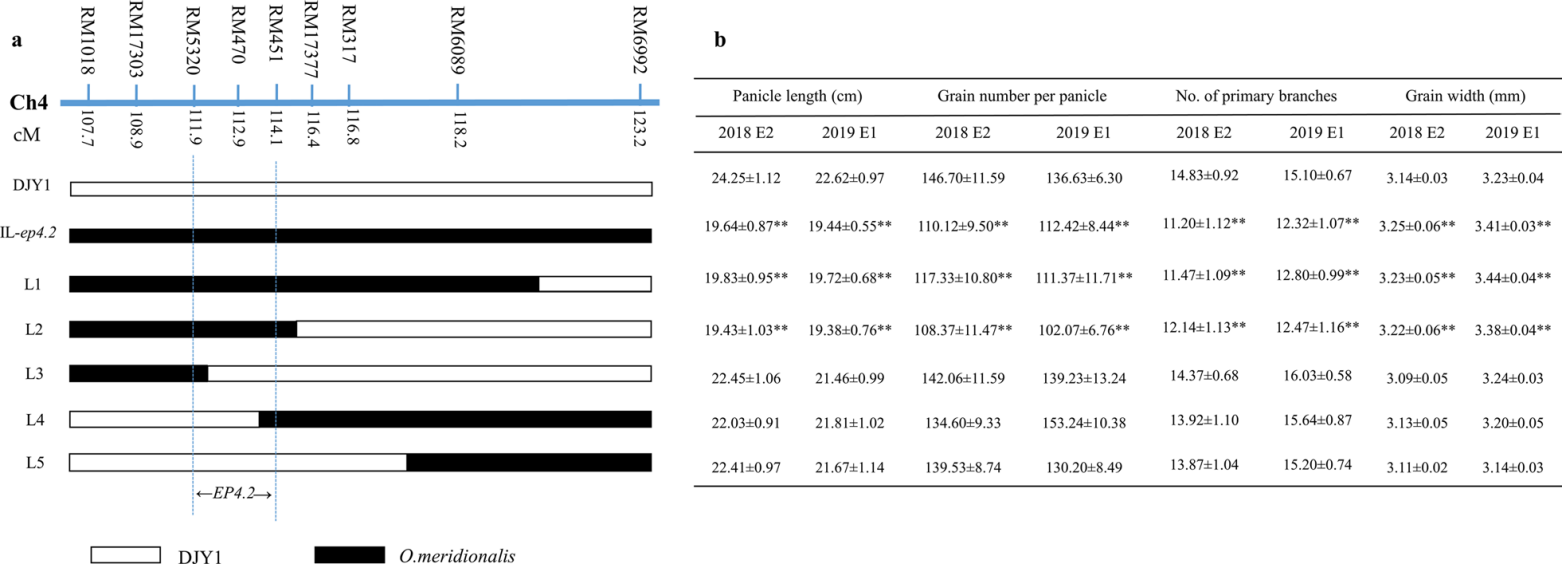
Mapping of the *EP4.2* locus to a 556 kb region between the marker RM5320 and RM451 in the long arm of chromosome 4.** a** Schematic map of five overlapping segment lines delimiting the mapping region for progeny traits analysis is presented by the different bars. And black and white bars referred to *O. meridionalis* and DJY1 homozygous alleles, respectively. **b** Traits comparison of panicle length, grain number per panicle, number of primary branch and grain width, respectively

For *EP4.2*, six homozygous substitution lines with different length overlapping segments were screened for phenotypic investigation in two seasons. The phenotypes of the overlapping segment lines exhibited that L1 and L2 harboring the introgression segments had significantly reduced PL, GNPP and NPB, while the other lines without the donor fragments in the mapping region showed the same phenotype as the recurrent parent DJY1. IL-*ep4.2* and L1, L2 had significantly increased GW than DJY1, too. By substitution mapping, *EP4.2* was mapped into a 556-kb region between RM5320 and RM451 (Fig. 2).

**Fig. 3:**
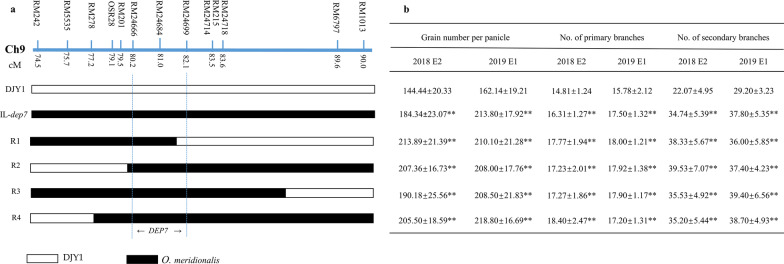
Mapping of the *DEP7* locus to a 472 kb region between the marker RM24666 and RM24699 in the long arm of chromosome 9. **a** Schematic map of four overlapping segment lines delimiting the mapping region for progeny traits analysis is presented by the different bars. And black and white bars referred to *O.meridionalis* and DJY1 homozygous alleles, respectively.** b** Traits comparison grain number per panicle, the number of primary and secondary branch, respectively

Using the same strategy, IL-*dep7* and four homozygous lines harboring the overlapping introgression segments were developed by continuous selfing and marker assisted selection. Phenotypic analysis of parents and four homozygous lines with different overlapping segments in two seasons showed that IL-*dep7* and four homozygous overlapping segment lines showed significantly increase in GNPP, NPB and NSB than those of DJY1. *DEP7* was narrowed down to a 472-kb region interval between RM24666 and RM24699 (Fig. 3).

**Fig. 4:**
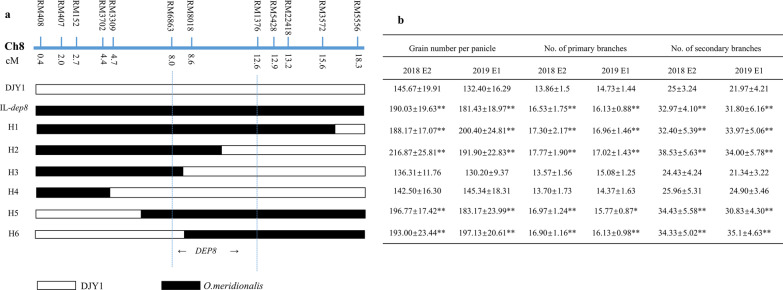
Mapping of the *DEP8* locus to an 1156 kb region between the marker RM6863 and RM1376 in the short arm of chromosome 8. **a** Schematic map of six overlapping segment lines delimiting the mapping region for progeny traits analysis is presented by the different bars. And black and white bars referred to *O.meridionalis* and DJY1 homozygous alleles, respectively. **b** Traits comparison of grain number per panicle, number of primary and secondary branch, respectively

Similarly, IL-*dep8* and six homozygous lines with overlapping segments of different length were developed by marker assisted selection, 12 polymorphic SSR markers were used for genotypic analysis. Phenotypic evaluation of parents and six homozygous lines with different length overlapping segments in two seasons showed that IL-*dep8*, H1, H2, H5 and H6 had significant increase in GNPP, NPB and NSB than those of DJY1, H3 and H4. Thus, *DEP8* was narrowed down to an 1157-kb region interval between RM6863 and RM1376 (Fig. 4).

### Various Genetic Effect of NIL*-ep4.2, *NIL*-dep7, *NIL*-dep8, *NIL*-dep1, *NIL*-er1*(t)*, *and NIL*-ep4* in DJY1 Background

To date, several QTL for panicle architecture, such as *DEP1* (Huang et al. 2009), *Er1*(t) (Zhou et al. 2008) and *EP4* (Zhang et al. 2015), have been identified from natural variations. *DEP1*, *Er1*(t) and *EP4* were identified from *O. sativa* spp. *japonica* cv. Shengnong 265, *O. glaberrima* and *O. glumaepatula*, respectively. Moreover, *Er1*(t) and *EP4* were also identified under the same genetic background of DJY1 as *EP4.2*, *DEP7* and *DEP8*. In this study, NIL-*dep1*, NIL-*er1*(t) and NIL-*ep4* in DJY1 genetic background were developed by marker-assisted selection based on previous research results (Zhou et al. 2008; Zhang et al. 2015). Then, six near isogenic lines only harboring the target introgression fragment were genotyped by high-density rice chip, and including NIL-*ep4.2*, NIL-*dep7*, NIL-*dep8*, NIL-*dep1*, NIL-*er1*(t) and NIL-*ep4* were used to investigate the QTL effect on the plant and panicle architecture. (Additional file 5: Figure S5).

**Fig. 5:**
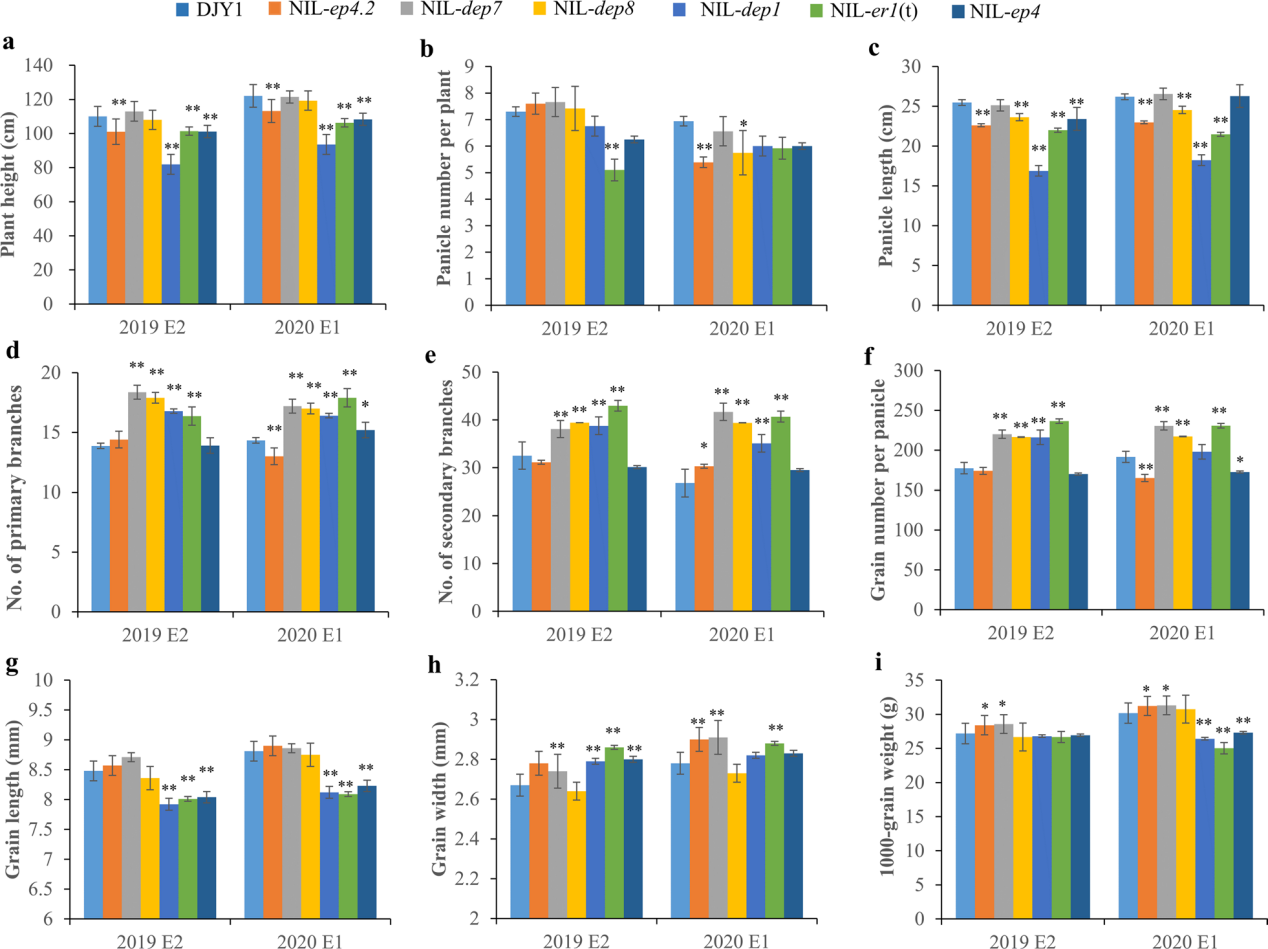
Agronomic traits comparison among *EP4.2*, *DEP7* and *DEP8* identified in this study with *DEP1*, *Er1*(t), *EP4* under DJY1 genetic background in two seasons (E2 of 2019 and E1 of 2020). **a**–**i** Analysis of significant differences in plant height, panicle number per plant, panicle length, number of primary branches, number of secondary branches, grain number per panicle, grain length, grain width and 1000-grain weight among DJY1, NIL-*ep4.2*, NIL-*dep7*, NIL-*dep8*, NIL-*dep1*, NIL-*er1*(t) and NIL-*ep4*. The asterisk * and **indicate significant difference from DJY1 at *p* < 0.05 and *p* < 0.01, respectively

Then, a comparison of agronomic traits among DJY1 and all near isogenic lines mentioned above was performed in two seasons. For PH, NIL-*dep7* and NIL-*dep8* showed no significant difference from DJY1, other NILs were significantly shorter than DJY1. Large phenotype variation in each NILs for PN was observed in the two seasons, in general, NIL-*dep1*, NIL-*er1*(t) and NIL-*ep4* exhibited fewer PN than DJY1. PL was significantly shorter in NIL-*ep4.2,* NIL-*dep8*, NIL-*dep1* and NIL-*er1*(t) compared with DJY1 under two seasons. Compared with DJY1, increased NPB of NIL-*dep7*, NIL-*dep8*, NIL-*dep1* and NIL-*er1*(t) were observed. In addition, the NPB in NIL-*ep4.2* and NIL-*ep4* showed remarkable difference, that might be associated with the environment factors. For NSB, NIL-*dep7*, NIL-*dep8*, NIL-*dep1* and NIL-*er1*(t) were significantly higher than DJY1. GNPP was significantly higher in NIL-*dep7*, NIL-*dep8* and NIL-*er1*(t) compared with DJY1. NIL-*dep1*, NIL-*er1*(t) and NIL-*ep4* exhibited shorter GL than DJY1. Wherease NIL-*ep4.2* and NIL-*er1*(t) showed wider grain width than DJY1. In addition, 1,000 grains weight (TGW) of NIL-*ep4.2* and NIL-*dep7* was significantly higher than recurrent parent DJY1 (Fig. 5).

Those results indicated that the three QTL identified from *O. meridionalis* had different effects on plant and panicle architecture traits compared with *dep1*, *er1*(t) and *ep4*. *O. meridionalis* alleles at *DEP7* and *DEP8* had positive effects on GNPP; the increasing of GNPP was mainly attributed to the enhanced NPB and NSB, meanwhile, the PH of *DEP7* and *DEP8* were not decrease significantly than that of DJY1 (Fig. 5), it is suggested that *DEP7* and *DEP8* were valuable QTL for increasing yield potential. *O. meridionalis* alleles at *EP4.2* conferred shorter plant height and wider grain width (Fig. 5). Thus, *EP4.2*, played an important role in the plant architecture and grain size.

### Pyramiding and Interaction Effect Among *EP4.2, DEP7 *and *DEP8*

In this study, *DEP7* and *DEP8* allele from *O. meridionalis* had positive effects on GNPP, NPB and NSB. *EP4.2* allele from the *O. meridionalis* acted a negative effect in PL, GNPP and NSB, whereas had positive additive effect on GW (Table 1). On the purpose of elucidating the cumulative effect and exploring the breeding value of *EP4.2, DEP7* and *DEP8*, the QTL pyramiding strategy was adopted. Three NIL-F_2_ segregation populations, *EP4.2* + *DEP7*, *EP4.2* + *DEP8*, and *DEP7* + *DEP8*, were developed to analyze the pairwise interaction effects of these three loci.

**Fig. 6:**
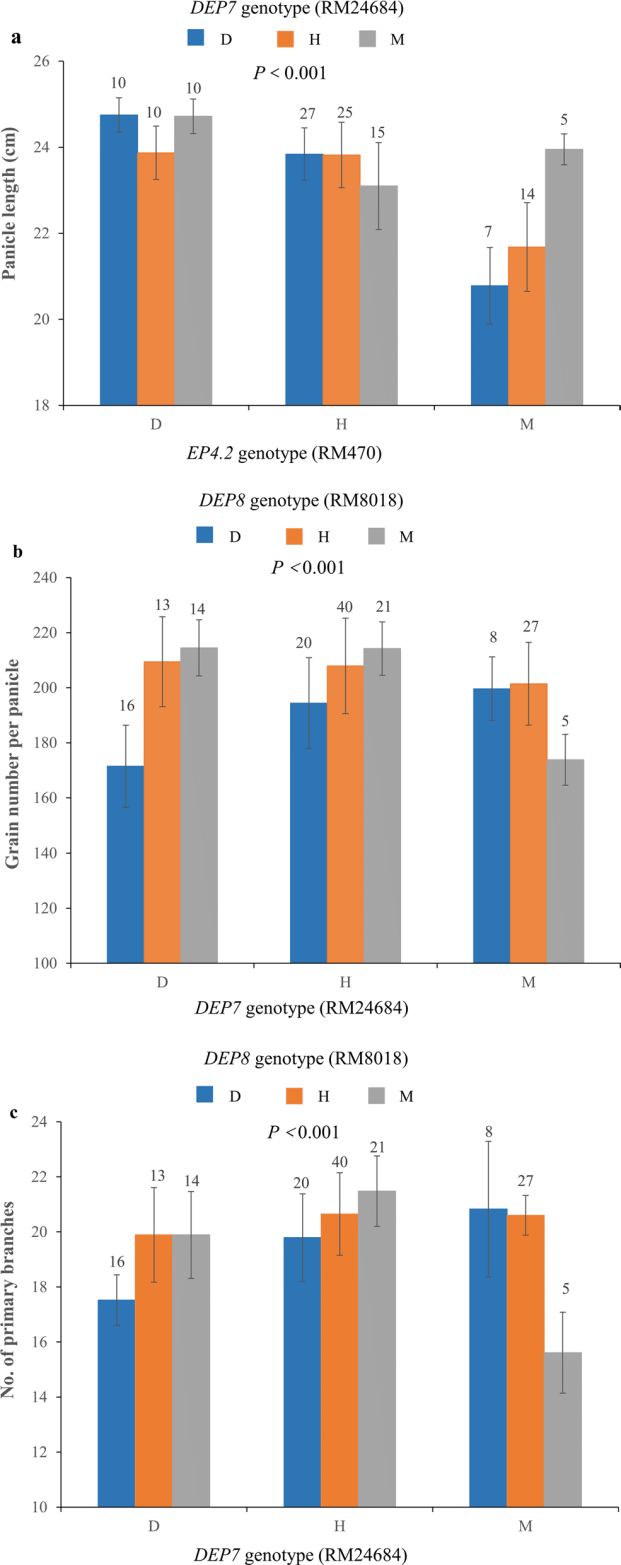
Gene–gene interaction analysis among *EP4.2*, *DEP7* and *DEP8*. **a** Effect of genetic interaction between *DEP7* and *EP4.2* on plant height in NIL-F_2_ population. *Lables below bars* refer to *EP4.2* genotypes; bar colors refer to *DEP7* genotypes (*blue* DJY1 homozygous; *orange* heterozygous; *gray* O. *merilionalis* homozygous). **b-c** Effect of genetic interaction between *DEP7* and *DEP8* on grain number per panicle and panicle length in NIL-F_2_ population. *Lables below bars* refer to *DEP7* genotypes; bar colors refer to *DEP8* genotypes (*blue* DJY1 homozygous; *orange* heterozygous; *gray* O. *merilionalis* homozygous). D, H, and M indicate homozygous for the DJY1 allele, heterozygosity, and homozygous for the *O. merilionalis* allele, respectively. *P*-value were calculated by two-way ANOVA

Since each NIL-F_2_ population consisted of all the nine types of genotypic combinations, the average value of PH, PL, NPB, NSB, GNPP, GL, GW and TGW among the nine classes were compared, respectively (Additional file 6: Table S1, Additional file 7: Table S2, Additional file 8: Table S3, Additional file 9: Table S4, Additional file 10: Table S5, Additional file 11: Table S6). The PL of three genotypes at *DEP7* showed no significant difference when *EP4.2* were heterozygous and homozygous for DJY1, while the PL of three genotypes of *DEP7* had significant difference when *EP4.2* harbored homozygous allele from *O. meridionalis*, which indicated that *EP4.2* and *DEP7* showed epistatic interaction effect on PL (Fig. 6a). Both of homozygous *DEP7* and *DEP8* alleles from *O. meridionalis* conferred the remarkable decreased GNPP and NPB (Fig. 6b–c). These results clearly indicated that *DEP7* interacted with *DEP8* to regulate the GNPP and NPB.

The QTL pyramided lines carrying two QTL, *EP4.2* + *DEP7*, *EP4.2* + *DEP8*, and *DEP7* + *DEP8*, were chosen from the NIL-F_2_ populations to explore the breeding value. The PH, PL, GNPP, NPB and NSB were investigated in DJY1, NIL-*ep4.2*, NIL-*dep7*, NIL-*dep8*, NIL-*ep4.2* + *dep7*, NIL-*ep4.2* + *dep8*, and NIL-*dep7* + *dep8*.

**Fig. 7:**
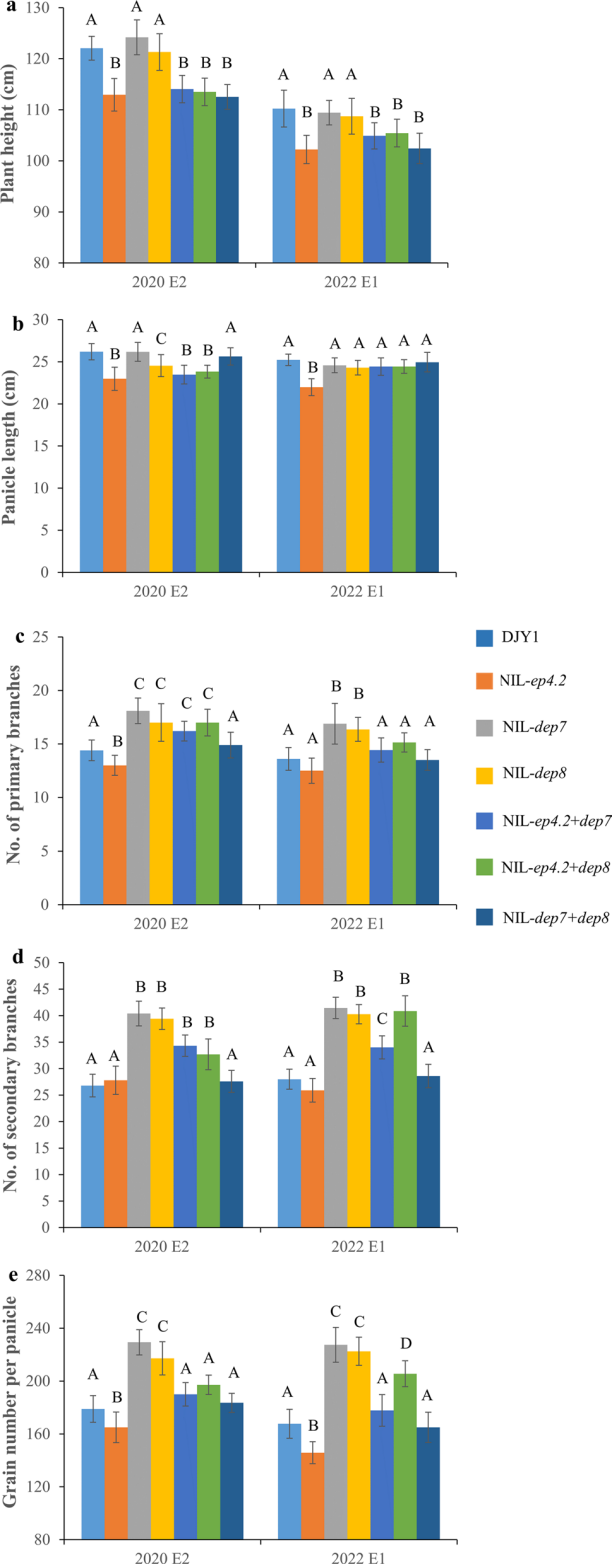
Performance of the recurrent parent DJY1, and six QTL-NILs for plant and panicle architecture traits. **a**–**e** Analysis of significant differences in plant height, panicle length, number of primary branches, number of secondary branches and grain number per panicle among DJY1, NIL-*ep4.2*, NIL-*dep7*, NIL-*dep8*, NIL-*ep4.2* + *dep7*, NIL-**ep4.2** + *dep8* and NIL-*dep7* + *dep8*. Capitals represent significant difference at 0.01 level

Compared with NIL-*ep4.2*, there was no significant difference in PH in three pyramided lines, NIL-*ep4.2* + *dep7*, NIL-*ep4.2* + *dep8*, NIL-*dep7* + *dep8*, however, three pyramided lines showed significantly shorter stature than that of NIL-*dep7* and NIL-*dep8* (Fig. 7). Meanwhile, the PL, GNPP, NPB and NSB of NIL-*ep4.2* + *dep7*, NIL-*ep4.2* + *dep8* were significantly increased than those of NIL-*ep4.2*, but decreased in comparison to NIL-*dep7* and NIL-*dep8*. The PL of NIL-*dep7* + *dep8* showed no difference from NIL-*dep7* and NIL-*dep8*. For GNPP, NPB and NSB, NIL-*dep7* + *dep8* showed significantly decreased compared with NIL-*dep7* and NIL-*dep8*. Compared with the NIL-*dep7* and NIL-*dep8*, PH, and GNPP of the pyramided lines were reduced. Those results further confirmed that three QTL played an important role in the improvement of plant and panicle architecture.

## Discussion

### *EP4.2, DEP7, DEP8 *from *O. meridionalis* Should be the New Allele for Panicle Architecture Improvement

In the present study, three QTL for panicle architecture were identified from *O. meridionalis*, named *EP4.2*, *DEP7* and *DEP8*, respectively. We compared the three QTL mapping results co-localized with previously reported QTL for similar panicle architecture traits, according to the source of alleles and physical positions of mapping interval markers (Table 2). *EP4.2* was mapped into a 556-kb region between RM5320 and RM451 on the long arm of chromosome 4, the *O. meridionalis* allele at *EP4.2* had positive effect on GW and negative effect on PL, GNPP, and NPB. Two QTL overlapping with the *EP4.2* region were identified, *qGN4-1* was detected from *O. sativa* between the marker interval RM2441 and HvSSR04-49, increasing grain number per panicle by increasing primary and secondary branches (Deshmukh et al. 2010). The *qPND-4* for panicle neck diameter (PND) was mapped into the interval between SSR marker RM241 and RM349, the chromosomal region also hosting QTL for seven panicle traits including panicle number, spikelet number per panicle, grain number per panicle, spikelet density, panicle length, primary and secondary branches. The allele from ‘IRAT109’ increased panicle neck diameter, spikelet number per panicle, grain number per panicle, spikelet density, panicle length, primary and secondary branches, while decreased the panicle number (Zou et al. 2005; Liu et al. 2008). *qGN4-1* and *qPND-4* gave the differnent phenotype from *EP4.2*.

**Table 2:** Comparison of the present identified QTL with previously reported QTL in the similar region

QTL	Interval (kb)	Interval length (kb)	Overlapped interval reported previously					
			QTL	Trait	Genomic region (kb)	Interval length (kb)	Donor species	Reference
*EP4.2*	27,988.3–28,544.7	556.4	*qGN4-1*	Grains per panicle	28,020.9–30,715.8	2694.9	*O. sativa*	Deshmukh et al. (2010)
			*qPND-4*	Panicle neck diameter	27,016.0–32,718.5	5702.5	*O. sativa*	Liu et al. (2008)
			*qPL-4*	Panicle length	28,248.9–28,544.7	295.8		
			*qPBN-4*	Primary branches	28,544.7–29,219.4	674.7		
			*qSBN-4*	Secondary branches	30,991.5–32,718.5	1727		
			*qSPD-4*	Spikelet density	30,991.5–32,718.5	1727		
			*qGNP-4*	Grains per panicle	29,219.4–30,991.5	1772.1		
			*qPN-4*	Panicles per plant	29,219.4–30,991.5	1772.1	*O. sativa*	Zou et al. (2005)
			*qSN-4*	Spikelets per panicle	29,219.4–30,991.5	1772.1		
*DEP8*	2006.0–3162.6	1156.6	*yldp8.1*	Yield per plant	2109.5–20,645.6	18,536.1	*O. nivara*	Kaladhar et al. (2008)
			*yld8.3*	Plot yield	2109.5–8310	6200.5	*O. rufipogon*	Marri et al. (2005)
			*yld8*	Grain yield	2109.6		*O. rufipogon*	Gaikwad et al. (2014)
*DEP7*	20,067.0–20,539.0	472	*spp9.1 gpp9.1* *pl9.1 yld9.1* *gw9.1*	Spikelets per panicle Grains per panicle Panicle length Yield per plant Grain width	18,642.2–22,419.8	3777.6	*O. rufipogon*	Thomson et al. (2003)
			*pl9.1* *gw9.1* *yldp9.1*	Panicle lengh Grain weight Yield per plant	18,642.2–22,419.8	3777.6	*O. rufipogon*	Marri et al. 2005
			*sn9.1 gn9.1 den9.1 pl9.1 yd9.1* *gw9.1*	Spikelets per panicle Grains per panicle Panicle density Panicle length Yield per plant Grain width	20,904.1–20,941.5	37.4	*O. rufipogon*	Xie et al. (2008)

*DEP7* was located in the region between RM24666 and RM24699 on the chromosome 9, enhancing GNPP by increasing the NPB and NSB. It means that the allele from *O. meridionalis* has the positive effect on GNPP, NPB and NSB. Meanwhile, *DEP7* did not affect PH and PL. Several QTL control panicle architecture were reported in similar chromosome intervals from *O. rufipogon* (Thomson et al. 2003; Marri et al. 2005; Xie et al. 2008) (Table 2). However, most of these alleles from *O. rufipogon* have positive effect, except for a allele control grain width. It is different from our results. *DEP8* was mapped into the region between RM6863 and RM1376 on the short arm of chromosome 8, which increased GNPP due to more NPB and NSB. In the overlapping region with *DEP8*, *yldp8.1*, *yld8* and *yld8.3* for grain yield were detected from *O. nivara* and *O. rufipogon*, respectively (Marri et al. 2005; Kaladhar et al. 2008; Gaikwad et al. 2014). However, there are no more information to explain these QTL how to affect the yield by influencing yield component factors.

Considering the different phenotype and there are no QTL about panicle architecture reported from the cross between *O. sativa* and *O. meridionalis*, *EP4.2*, *DEP7* and *DEP8* were firstly confirmed as stable QTL controlling panicle architecture from *O. meridionalis*.

### Substitution Mapping is an Effective Method to Identify Quantitative Trait Loci

Most important agronomic traits including yield components are inherited in a quantitative manner and generally controlled by a number of major and minor quantitative trait loci (QTL). Many major QTL for yield components were cloned (Ashikari et al. 2005; Huang et al. 2009; Qiao et al. 2021). However, only a few minor QTLs were cloned due to the easily affected by environment and genetic background (Hori et al. 2016; Chen et al. 2018; Chan et al. 2021). Some reports indicated that the minor QTL can be detected stably when the genetic background is highly homozygous (Chen et al. 2014; Wang et al. 2019). Therefore, minor QTL can be verified and decomposed, and even further fine mapped and cloned under high homogeneity genetic background.

Near isogenic lines (NILs) have been proven to be an effective resource for QTL validation and fine mapping, owing to the elimination of genetic background noise (Bai et al. 2012). By developing multiple NILs covering different interval segments, substitution mapping can be employed to effectively dissect the QTL (Paterson et al. 1990). Using the method of substitution mapping, minor QTL for 1,000 grains weight including *qTGW1.2a* and *qTGW1.2b* were fine-mapped, and the candidate gene of *qTGW1.2b* was confirmed (Chen et al. 2014; Wang et al. 2019)*.* Similary, the QTL of aerobic adaptation, *qAER1, qAER3* and *qAER9*, were verified and fine-mapped (Xu et al. 2020). Furthermore, a small number of QTLs for heading date were validated using substitution mapping, too (Xie et al. 2008; Yan et al. 2011).

In present study, IL-*dep7*, IL-*dep8*, and recurrent parent DJY1 showed significant difference for GNPP, SD, NPB and NSB, respectively. However, QTL detection for two of four traits in two BC_6_F_2_ populations (2016H2E273, 2016H3E3182) with low LOD values (2.38 and 2.41, 2.46 and 3.30, respectively). Substitution mapping using homozygous lines that harbored the different length overlapping segments from *O. meridionalis* was used to validate the QTL and delimit the mapping region further. By comparing the phenotypes of several homozygous lines that harbored the different length overlapping segments, *EP4.2*, *DEP7*, *DEP8* were mapped into the regions about 556-kb, 472-kb, 1156-kb between SSR markers RM5320 and RM451, RM24666 and RM24699, RM6863 and RM1376 on the chromosome 4, 9, 8, respectively. The mapping region was significantly shorter than those of QTL mapping. Therefore, substitution mapping and progeny validation are an effective method to validate quantitative trait loci mapping results.

### Introgression and Exploration of QTL for Panicle Architecture from Wild Relatives of Rice

Rice is one of the most important staple crops for almost half of the global population. The genetic diversity of cultivated rice was gradually reduced in the process of domestication (Huang et al. 2012; Chen et al. 2019), the narrow genetic basis led to yield bottleneck in rice breeding (Tanksley and Mccouch 1997). The abundant genetic variation in the wild relatives of rice is an excellent gene pool for genetic improvement of cultivated rice. Although the yield of wild relatives is lower than cultivated rice, many studies found that wild relatives still carried the yield related QTL for improving cultivated rice (Xiao et al. 1996, 1998; Thomson et al. 2003). In the recent years, introgression and exploration of QTL for yield-related traits from wild relatives have made great progress in rice breeding. As the wild ancestor of Asian cultivated rice, *O. rufipogon* has been extensively used for improving yield related traits of Asian cultivated rice (Xiao et al. 1996, 1998; Xie et al. 2008). Apart from *O. rufipogon*, several yield-enhancing alleles were successfully introduced from *O. nivara* (Kaladhar et al. 2008; Ma et al. 2016), *O. grandiglumis* (Yoon et al. 2006), *O. glumaepatula* (Bhatia et al. 2018; Zhao et al. 2019), *O. minuta* (Li et al. 2020), *O. glaberrima* (Li et al. 2004), *O. barthii* (Bessho-Uehara et al. 2017; Zhao et al. 2019), *O. longistminata* (Gichuhi et al. 2016), *O. meridionalis* (Varma et al. 2012; He et al. 2017) and *O. meyeriana* (Chen et al. 2012). Recently, Zhang et al. (2022) developed agronomic introgression lines with all AA genome species as multiple donors, and detected 85 loci controlling GL and GW from different AA genome donors. Although hundreds of QTL for yield traits were detected from wild species of rice, only few of them were used for in-depth study. Xiao et al. (1998) identified 68 QTL for 12 yield related traits in interspecific BC_2_ testcross population from *O. rufipogon*, the *O. rufipogon* alleles at *yld1.1* and *yld2.1* increased grain yield to 18% and 17%, respectively. These two QTL were later introgressed into the elite restorer lines Mnghui 63, 93-11 and Ce64-7 through marker assisted selection and significant yield improvement was observed (Luo et al. 2001).

In current study, two accessions of *O. meridionalis* were used as donors to develop introgression lines (ILs) in DJY1 background. Three QTL influencing panicle architecture traits were identified and validated. The three QTL are beneficial gene resources for molecular breeding in rice improvement.

## Materials and Methods

### Development of Introgression Lines for Panicle Architecture

A total of eight accessions of *O. meridionalis* as donor parents were crossed with Dianjingyou 1 (DJY1), a *japonica* variety bred by Yunnan Academy of Agricultural sciences (YAAS), to develop agronomic trait introgression lines (ILs) (Zhang et al. 2022). In this study, based on phenotypic evaluation, three BC_5_F_9_ ILs with dense and/or erect panicle (DEP) were developed from two accessions (Acc.104498 and Acc.105279) of *O. meridionalis* by five times backcross and multiple selfing. A total of 404 simple sequence repeat (SSR) markers covering the whole rice genome were used for surveying polymorphism between ILs and the recurrent parent DJY1. Only 6 polymorphic markers on chromosome 4, 10 polymorphic markers on chromosome 9 and 12 polymorphic markers on chromosome 8 were detected between DJY1 and the three ILs, respectively. No polymorphic markers were detected on the other chromosomes, indicating that each IL carried only one chromosomal fragment from *O. meridionalis.* Then, the three ILs were named as IL-*ep4.2*, IL-*dep7* and IL-*dep8*, respectively.

All plant materials were planted with 15 cm between plants and 25 cm between rows in the paddy field at the Winter Breeding Station, Yunnan Academy of Agricultural Sciences located in Sanya, Hainan Province, P. R. China. Field management followed essentially the ordinary agricultural practice in this area.

### Mapping Populations Development and QTL Analysis

For QTL mapping, a BC_6_F_2_ population from Acc. 104,498 consisting of 208 plants derived from a cross between the IL-*dep7* and DJY1, was cultivated in Winter rice growing season (November–April, E2) of 2015. Meanwhile, two BC_6_F_2_ populations from Acc.105279 consisting of 298 and 261 individuals derived from the IL-*ep4.2* and IL-*dep8* crossed with DJY1, respectively, were planted in Summer rice growing season (May–October, E1) of 2016. Three BC_6_F_2_ populations were genotyped using the polymorphic markers between ILs and DJY1, respectively. The panicle architecture traits including panicle length (PL, cm), grain number per panicle (GNPP), spikelet density (SD), the number of primary and secondary branches (NPB and NSB), grain length (GL, mm), grain width (GW, mm) of all individuals of BC_6_F_2_ populations and parents were investigated from three primary panicles of each plant after harvested. Spikelet density (SD) was evaluated by the ratio of spikelet numbers to panicle length. All panicles were dried naturally after harvesting and stored at least one month before testing. Correlation coefficient analysis was performed using R 3.5.3 (R package corrplot) (Murdoch and Chow 1996). QTL was detected with composite interval mapping by using WinQTL Cartographer v2.0 (Wang et al. 2012). The logarithm of the odds thresholds were calculated based on 1,000 permutations (P < 0.05) and used to declare a putative QTL.

### Development Overlapping Segment Lines for QTL Verification

Recombinant individuals of BC_6_F_2_ populations were self-fertilized to develop BC_6_F_4_ and BC_6_F_5_ homozygous overlapping segment lines to validate QTL mapping and precisely delimit their location by marker-assisted selection (MAS). The overlapping segment lines were planted in E2 of 2018 and E1 of 2019 by randomized complete block design with three replications, and twenty plants from each line from the middle of each plot were sampled. Plant height (PH, cm), panicle number per plant (PN) were investigated in the field at the mature stage. PL, GNPP, NPB, NSB, GL, GW and 1,000 grain weight (TGW, g) were investigated from three primary panicles per plant after harvested. All panicles were dried naturally after harvesting and stored at least one month before testing.

### Development of six NILs with Panicle Architecture Loci

NIL-*dep1*, NIL-*er1*(t) and NIL-*ep4* were developed based on previous study (Zhou et al. 2008; Zhang et al. 2015). Three homozygous overlapping segment lines, L2, R2 and H2, were genotyped by rice 6 K SNP-chip, the individual with the most similar genotypes to the recurrent parent DJY1 were selected as NIL-*ep4.2*, NIL-*dep7* and NIL-*dep8*. The NILs were planted in E2 of 2019 and E1 of 2020. The investigation methods of phenotype were described as Sect.  4.3. The least significant difference (LSD) method was used to compare the mean values of the tested traits for six QTL-NILs and recurrent parent DJY1.

### Genetic Interaction Analysis Among *EP4.2, DEP7 *and* DEP8*

To analyze the genetic interactions among the QTL detected in our study, NIL-*ep4.2,* NIL-*dep7* and NIL-*dep8* were crossed in pairs. NIL-F_2_ individuals were genotyped using RM470, RM24684 and RM8018 tightly linked with *EP4.2*, *DEP7* and *DEP8*, respectively. For the NIL-F_2_ populations, two-way analysis of variance (AVOVA) was conducted to test the main and epistatic effects. Duncan’s multiple range test was used to examine the phenotypic differences among nine genotypic groups. The analysis was performed using the SPSS Statistics 20 (IBM, NK, USA).

### DNA Extraction and PCR Amplification

DNA was extracted from the leaf of each plant using simple DNA extraction method (Edwards et al. 1991). PCR was performed in a reaction volume 10 µl containing 10 ng of template DNA, 0.2 µM of each forward and reverse primers, 5 µL 2 × *taq* PCR StarMix (Genestar Company, Beijing, China). PCR amplication was carried out as follows: 5 min at 94℃ for initial denaturation, followed by 32 cycles of 30 s at 94℃, 40 s at 55℃, 40 s at 72℃, and 5 min at 72℃ for a final extension. PCR amplification products were detected on 8% nondenaturing polyacrylamide gels using silver staining method (Shi et al. 2005).

## Conclusions

This study aims to explore QTL for panicle architecture that can be used in genetic study and breeding utilization from *O. meridionalis*. Three QTL responsible for panicle architecture traits were validated by substitution mapping. We compared the yield-related traits among *EP4.2, DEP7,* and *DEP8* with three published panicle architecture QTL under DJY1 genetic background. The results indicated that *DEP7* and *DEP8* conferred yield-enhancing potential while *EP4.2* had effect on plant and panicle architecture-improving potential. These results will lay the foundation for using panicle architecture QTL in rice breeding and provide a basis for the gene cloning.

## Supplementary Information

Additional file 1: Figure S1. Frequency distribution of phenotypes for 6 panicle architecture traits in 2016H3E3180 population from a cross between IL-ep4.2 and DJY1.

Additional file 2: Figure S2. Frequency distribution of phenotypes for 6 panicle architecture traits in 2016H2E273 population derived from a cross between IL-dep7 and DJY1.

Additional file 3: Figure S3. Frequency distribution of phenotypes for 6 panicle architecture traits in 2016H3E3182 population derived from a cross between IL-dep8 and DJY1.

Additional file 4: Figure S4. Correlation coefficient analysis for panicle architecture traits in three BC6F2 populations. a-c Correlation coefficient analysis for panicle architecture traits in populations derived from IL-ep4.2 and DJY1, IL-dep7 and DJY1, IL-dep8 and DJY1, respectively.

Additional file 5: Figure S5. Graphical genotype for NIL-ep4.2, NIL-dep7, NIL-dep8, NIL-dep1, NIL-er1, and NIL-ep4. White bar indicated chromosome segments derived from DJY1, red bar indicated chromosome regions from donors.

Additional file 6: Table S1. Mean performance of the plant height and panicle architecture trait of the nine genotypes in NIL-F2 population from the cross between NIL-ep4.2 and NIL-dep7.

Additional file 7: Table S2. Mean performance of the plant height and panicle architecture trait of the nine genotypes in NIL-F2 population from the cross between NIL-ep4.2 and NIL-dep8.

Additional file 8: Table S3. Mean performance of the plant height and panicle architecture trait of the nine genotypes in NIL-F2 population from the cross between NIL-dep7 and NIL-dep8

Additional file 9: Table S4. Interaction analysis between EP4.2 and DEP7 in NIL-F2 population

Additional file 10: Table S5. Interaction analysis between EP4.2 and DEP8 in NIL-F2 population

Additional file 11: Table S6. Interaction analysis analysis between DEP7 and DEP8 in NIL-F2 population

## Data Availability

The data sets supporting the results of this article are included within the article and its additional files.

## Abbreviations

QTL: Quantitative trait locus
*Gn1a*: *Grain number 1a*
*IPA1*: *Ideal plant architecture 1*
*DEP1*: *Dense and erect panicle 1*
*GNP1*: *Grain number per panicle 1*
*TAW1*: *TAWAWA1*
*GS5*: *Grain size 5*
*NIL*: Near isogenic line
PH: Plant height
PN: Panicle number per plant
PL: Panicle length
GNPP: Grain number per panicle
SD: Spikelet density
NPB: Number of primary branches
NSB: Number of secondary branches
GL: Grain length
GW: Grain width
TGW: 1,000 Grains weight
