# Supplementary figures and images for: Three QTL from Oryza meridionalis Could Improve Panicle Architecture in Asian Cultivated Rice
Source: Rice (N Y). 2023 May 2;16:22. doi: 10.1186/s12284-023-00640-5 (PMC10154444; doi:10.1186/s12284-023-00640-5)

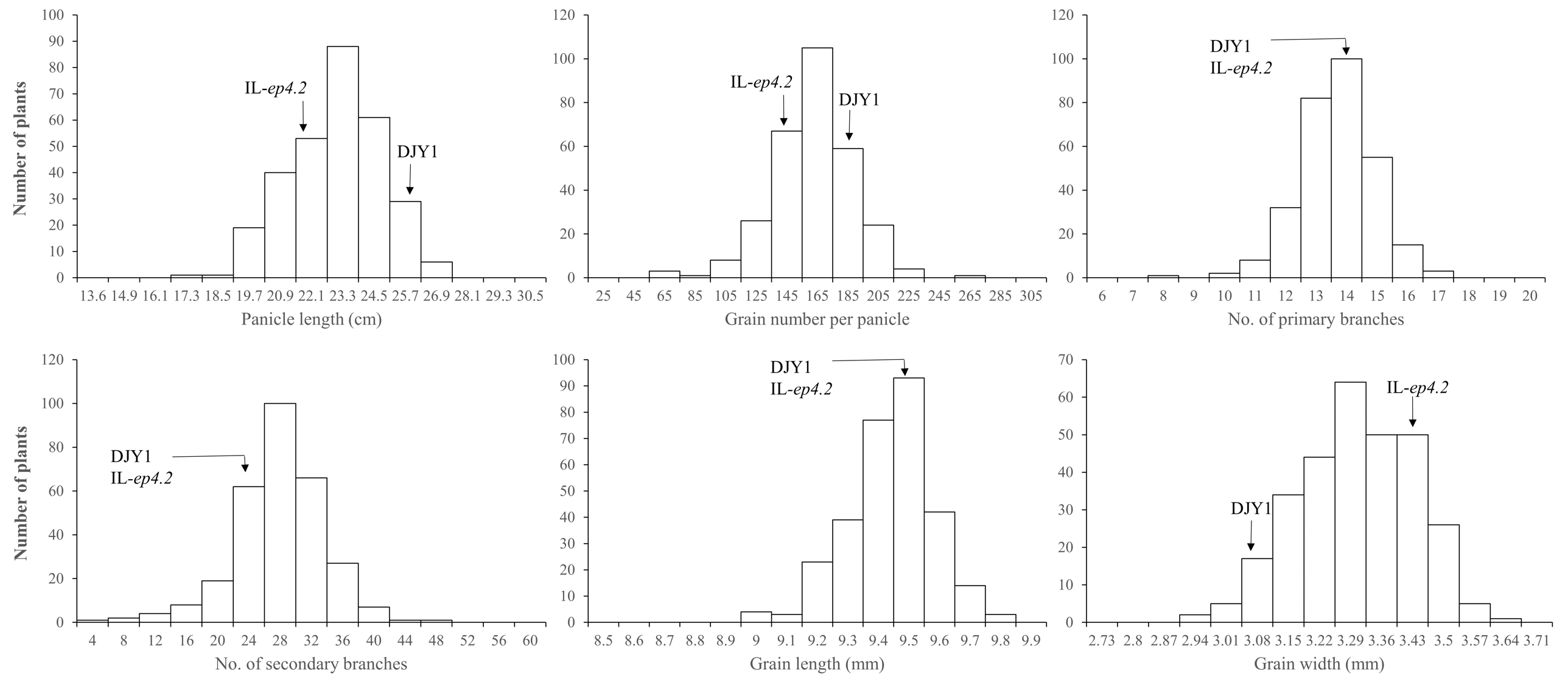

Supplement: Supplementary file 1 — Additional file 1: Figure S1. Frequency distribution of phenotypes for 6 panicle architecture traits in 2016H3E3180 population from a cross between IL-ep4.2 and DJY1. [file 12284_2023_640_MOESM1_ESM.png]

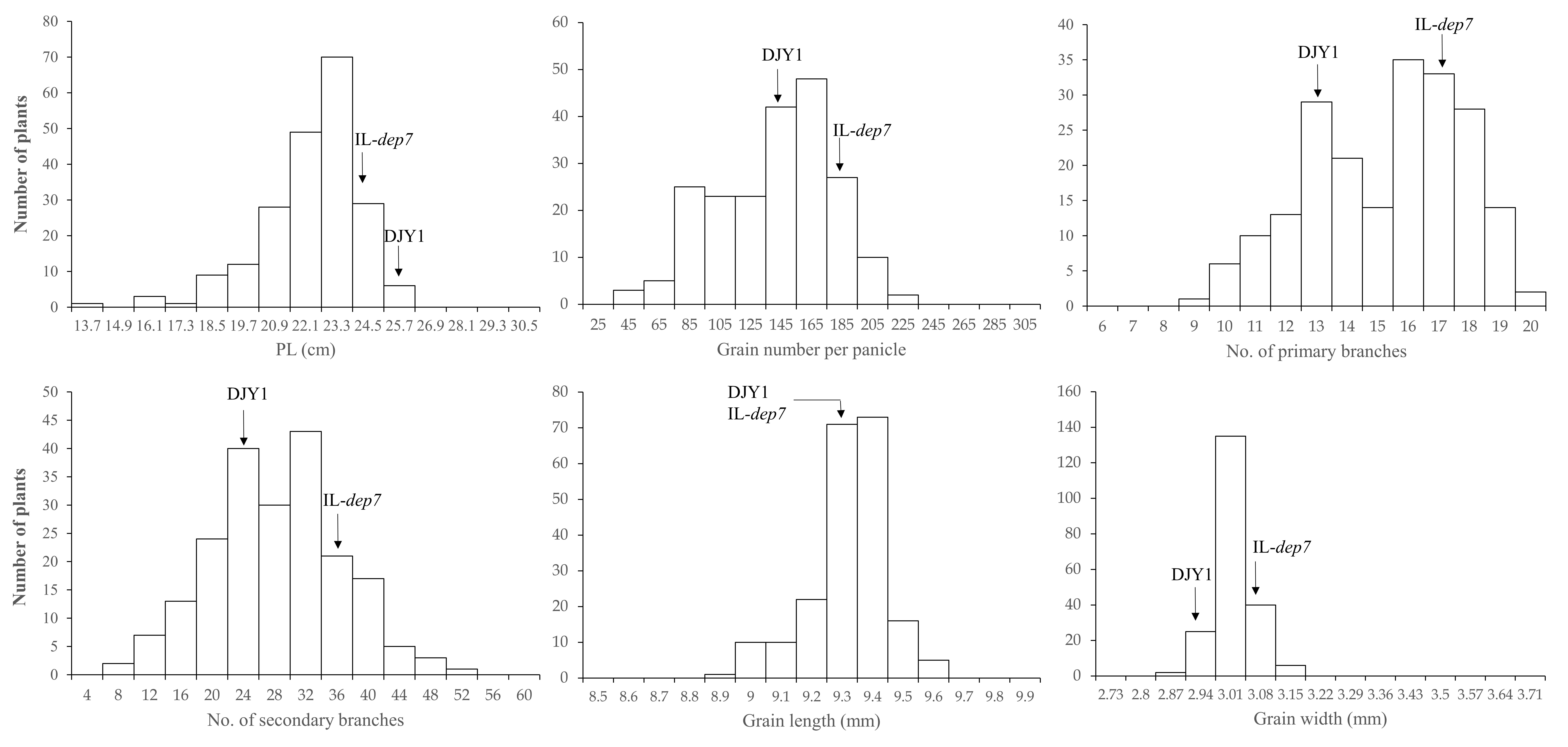

Supplement: Supplementary file 2 — Additional file 2: Figure S2. Frequency distribution of phenotypes for 6 panicle architecture traits in 2016H2E273 population derived from a cross between IL-dep7 and DJY1. [file 12284_2023_640_MOESM2_ESM.png]

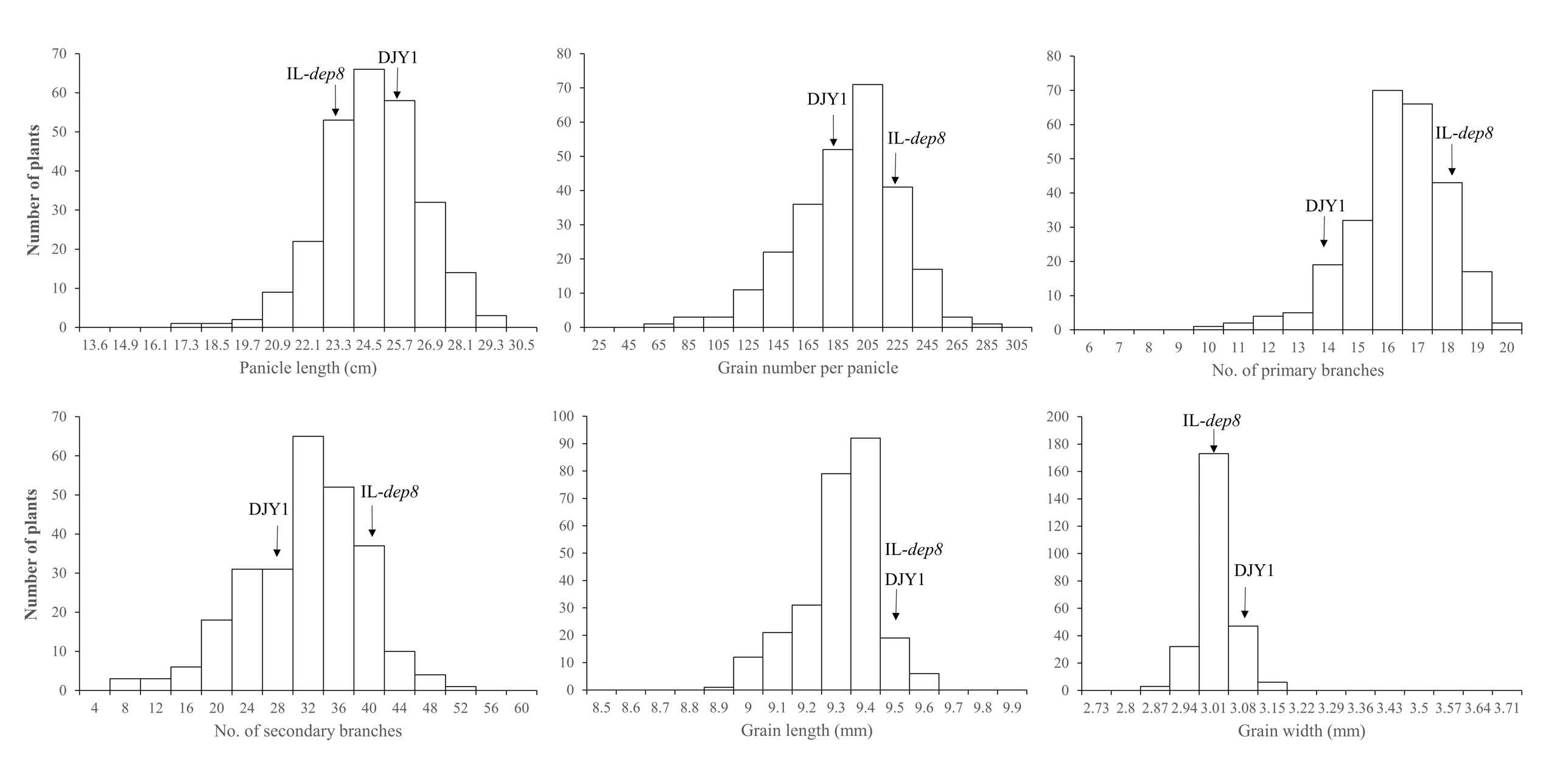

Supplement: Supplementary file 3 — Additional file 3: Figure S3. Frequency distribution of phenotypes for 6 panicle architecture traits in 2016H3E3182 population derived from a cross between IL-dep8 and DJY1. [file 12284_2023_640_MOESM3_ESM.png]

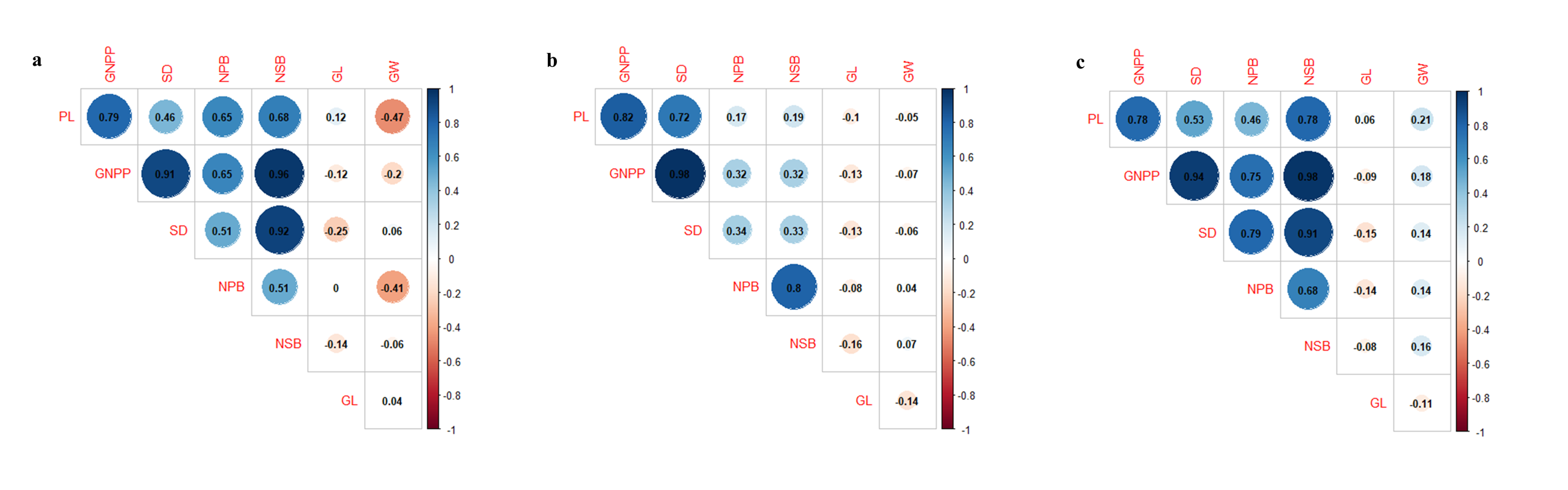

Supplement: Supplementary file 4 — Additional file 4: Figure S4. Correlation coefficient analysis for panicle architecture traits in three BC6F2 populations. a-c Correlation coefficient analysis for panicle architecture traits in populations derived from IL-ep4.2 and DJY1, IL-dep7 and DJY1, IL-dep8 and DJY1, respectively. [file 12284_2023_640_MOESM4_ESM.png]

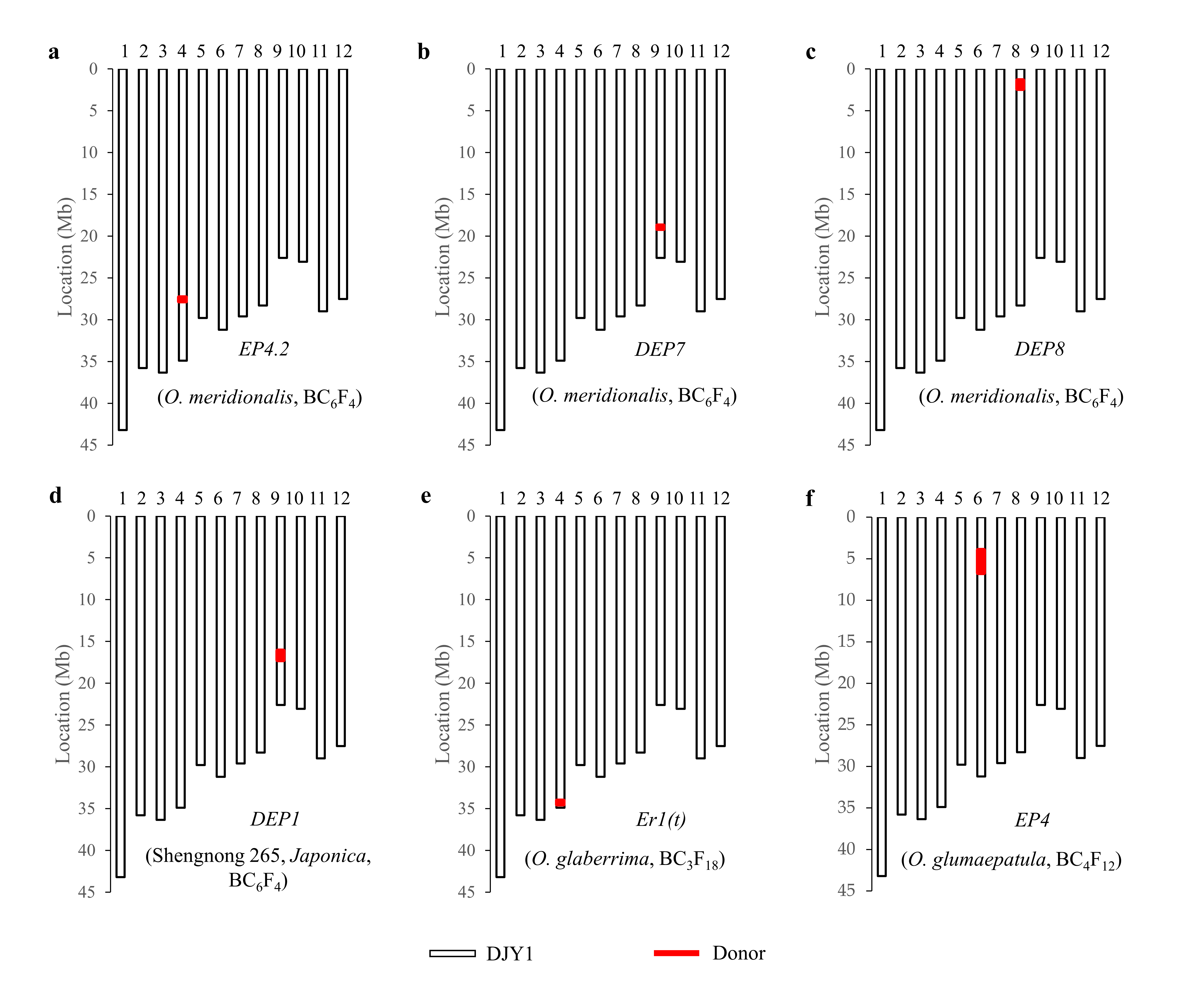

Supplement: Supplementary file 5 — Additional file 5: Figure S5. Graphical genotype for NIL-ep4.2, NIL-dep7, NIL-dep8, NIL-dep1, NIL-er1, and NIL-ep4. White bar indicated chromosome segments derived from DJY1, red bar indicated chromosome regions from donors. [file 12284_2023_640_MOESM5_ESM.png]
